# Ventilation-perfusion Scan: A Functional Imaging Approach to Regional Lung Disease in Primary Ciliary Dyskinesia

**DOI:** 10.2174/0118743064429529260119050836

**Published:** 2026-02-02

**Authors:** Gabriel Román-Ríos, Gabriel Rosario-Ortiz, Marcos J. Ramos-Benitez, Ricardo A. Mosquera, Wilfredo De Jesús-Rojas

**Affiliations:** 1 Department of Basic Sciences, Ponce Health Sciences University, Ponce, PR00716, USA; 2 Department of Pediatrics, McGovern Medical School, University of Texas Health Science Center at Houston, Houston, TX77030, USA

**Keywords:** Ciliary dyskinesia, V/Q scan, *RSPH4A*, Lung disease, Bronchiectasis, Chest CT imaging

## Abstract

**Introduction:**

As a rare genetic disorder defined by defective ciliary motility, Primary Ciliary Dyskinesia (PCD) leads to chronic respiratory complications. A founder variant in the *RSPH4A* [c.921+3_921+6delAAGT] gene, common in Puerto Rico, impairs ciliary function and contributes to progressive lung disease. Today, the clinical value of ventilation/perfusion (V/Q) scans in PCD has not been thoroughly investigated as well as pulmonary function tests (PFTs) and chest imaging. To explore the role and clinical utility of V/Q scans in PCD.

**Methods:**

Retrospective evaluation of pulmonary function tests, chest imaging, and Tc-99m DTPA ventilation and Tc-99m MAA perfusion scans in ten patients with genetically confirmed *RSPH4A* -associated PCD.

**Results:**

Regional lung dysfunction was present in V/Q scans of patients with PCD. Age-related FEV_1_ decline and severity of bronchiectasis on chest imaging were depicted in V/Q scans.

**Discussion:**

These findings suggest that V/Q scans can provide additional functional and physiological information beyond that obtained from CT scans in patients with PCD. Furthermore, future longitudinal studies are needed to confirm their value for monitoring disease progression and guiding clinical care in PCD.

**Conclusion:**

V/Q scans may identify functional abnormalities in PCD and complement standard imaging and pulmonary function tests. These scans may serve as valuable tools for monitoring disease progression and informing clinical decision-making.

## 1 INTRODUCTION

Primary Ciliary Dyskinesia (PCD) is a rare, genetically heterogeneous disorder caused by defects in motile cilia, leading to impaired mucociliary clearance and chronic respiratory disease [ 1 ]. Neonatal respiratory distress, persistent wet cough, sinusitis, otitis media, and bronchiectasis are common findings of this disorder [ 2, 3]. PCD diagnosis can be a challenge, since it often requires a clinical history with a combination of different tools, like nasal nitric oxide measurements [4], ciliary ultrastructure assessment [5], high-speed videomicroscopy [6], and genetic testing [7]. A population-based pathogenic variant in the *RSPH4A* [c.921+3_921+6delAAGT] gene has been identified as a prevalent founder variant associated with PCD in Puerto Rico [8]. The intronic deletion in the *RSPH4A* gene affects the radial spoke head protein 4A, which is critical for proper ciliary beat function [9]. *RSPH4A*-associated PCD shows a progressive decline in pulmonary function. Over time, patients develop increasing evidence of airway damage, bronchiectasis, and hypoxemia, despite early supportive care [10]. Pulmonary function tests (PFTs) [11], impulse oscillometry [12] and chest Computed Tomography (CT) scans [13] are commonly used to monitor lung disease progression in PCD. However, there is a gap in knowledge regarding the role of nuclear imaging in PCD, particularly the ventilation/perfusion (V/Q) scan. Today, V/Q scans are used in cystic fibrosis, chronic obstructive pulmonary disease (COPD), and pulmonary embolisms to evaluate lung function and regional perfusion mismatches [14-16]. Information about the application in PCD remains limited. Understanding the anatomic and physiologic changes present on V/Q imaging may enhance PCD disease monitoring and improve clinical decisions. V/Q scans may predict the need for therapeutic escalation or transplant evaluation. Our communication tries to explore the medical relevance of V/Q scans in characterizing PCD pulmonary involvement, especially among our patients with *RSPH4A*-associated PCD in Puerto Rico.

## 2 MATERIALS AND METHODS

A retrospective review of clinical data from a cohort of ten ( *n* =10) patients from Puerto Rico with PCD carrying the homozygous or compound heterozygous *RSPH4A* [c.921+3_921+6delAAGT] genetic variant was conducted. The ten patients were evaluated at the PCD center in Puerto Rico between 2019 and 2024. Inclusion criteria required a confirmed PCD genetic diagnosis and the availability of V/Q scans, PFTs, and chest imaging. Pulmonary function tests included spirometry (FEV_1_ ), which were performed according to ATS/ERS guidelines [ 17 ]. V/Q scans were reviewed as part of the comprehensive pulmonary evaluation at diagnosis to identify regional ventilation-perfusion mismatch. Tc-99m DTPA (diethylene-triamine-pentaacetate) aerosol for venti-lation imaging and Tc-99m macroaggregated albumin (MAA) for perfusion were used in all V/Q scans included in this study. All V/Q scans were completed at the same clinical center and with the same equipment. A board-certified nuclear medicine physician interpreted and reviewed all scans for the presence and distribution of V/Q mismatches or defects. All chest CT scans were analyzed to assess the presence and severity of bronchiectasis, and other structural abnormalities among different ages in axial and coronal views. Correlations and comparisons between imaging findings, age, and FEV_1_ were completed. The Institutional Review Board of Ponce Health Sciences University (IRB protocol #2309163682) approved this protocol on February 5, 2024.

## 3 RESULTS

### 3.1 Subjects Characteristics

A total of 10 patients ( *n* =10) with genetically confirmed PCD, all homozygous for the *RSPH4A* [c.921+3_ 921+6delAAGT] genetic variant, were evaluated with PFTs, chest CT scans, and V/Q scans. The cohort in this study included both pediatric ( *n* =5) and adult ( *n* =5) patients. Age ranged from 11 to 55 years, with 7 out of 10 patients being females. Table ****1**** shows patient character-istics, including age, sex, genotype, and infection status.

### 3.2 Pulmonary Function Tests (PFTs)

Preserved lung function (mean FEV_1_ : 62%) was present in pediatric patients, whereas adult patients demonstrated moderate to severe airflow limitation (mean FEV_1_ : 43%) in most cases. A 11-year-old male with *Burkholderia cepacia* had the lowest FEV_1_ (48%) among all pediatric patients.

### 3.3 Chest CT Scan

**Fig. (1):**
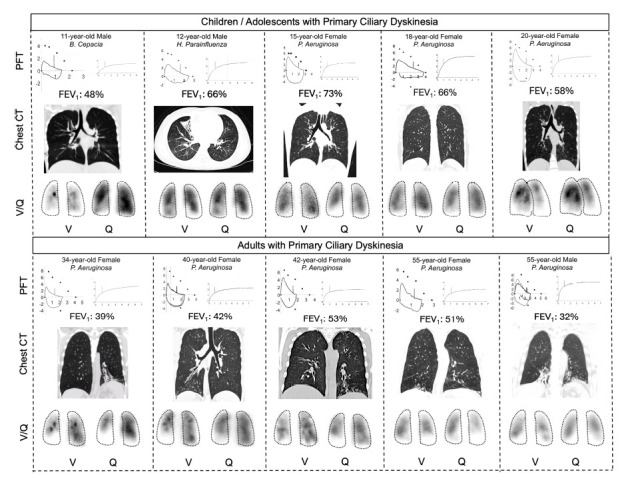
Pulmonary Function Testing, Chest CT Imaging, and Ventilation/perfusion (V/Q) Scans in Pediatric and Adult Patients with Primary Ciliary Dyskinesia (PCD) carrying the Homozygous *RSPH4A* [c.921+3_921+6delAAGT] Genetic Variant. Each panel includes FEV_1_ values, coronal or axial chest CT images, and posterior V/Q scan views. Tc-99m DTPA was used for ventilation, and Tc-99m MAA for perfusion. A progressive decline in lung function is observed across age groups, with worsening FEV_1_ values and increasingly severe bronchiectasis on CT scans. V/Q scans demonstrate a corresponding increase in ventilation–perfusion defects, particularly in the lower lobes.

The CT scans showed the presence of worsening bronchiectasis with age, mucus plugging, and airway wall thickening, with more severe abnormalities observed in adults (Fig. ****1**** ). All pediatric patients presented early structural changes in the lower lobes. The adults demons-trated widespread bilateral bronchiectasis and architec-tural distortion on CT scans.

### 3.4 Ventilation/Perfusion (V/Q) Scan

**Fig. (2):**
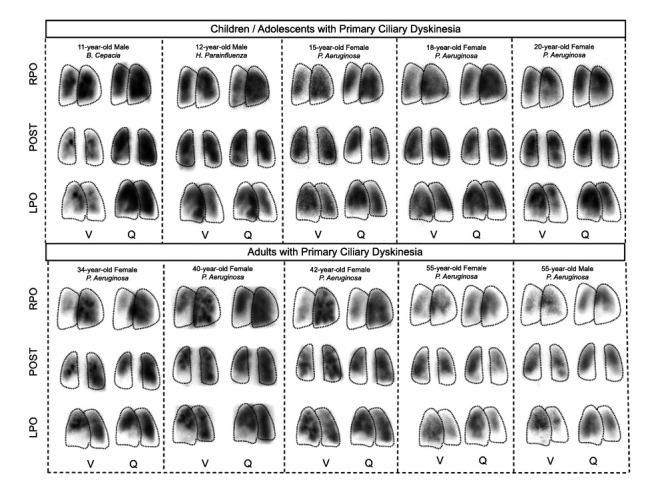
Ventilation/perfusion (V/Q) scans in Pediatric and Adult Patients with Primary Ciliary Dyskinesia (PCD) carrying the homozygous *RSPH4A* [c.921+3_921+6delAAGT] genetic variant. Representative planar V/Q scan images from children, adolescents, and adults with genetically confirmed PCD are shown in Anterior (ANT), Posterior (POST), Right Posterior Oblique (RPO), and Left Posterior Oblique (LPO) projections.

All patients with PCD demonstrated V/Q scans with mismatches (Fig. ****2**** ). Pediatric patients showed peripheral ventilation defects, with mostly preserved perfusion. Compared with children, adults showed pronounced mismatches. The extent of V/Q abnormalities increases with age, reductions in FEV_1_ , and radiographic changes on chest CT scans.

## 4 DISCUSSION

This study explores the role of complementary pulmonary function tests, chest CT scans, and V/Q scans in a genetically homogeneous PCD cohort to create a multidimensional view of disease progression, using V/Q mismatch as a possible marker of worsening lung function. A significant V/Q mismatch as a hallmark of lung dysfunction in Puerto Rican patients with PCD carrying the homozygous *RSPH4A* [c.921+3_921+6delAAGT] genetic variant was depicted in this study. The cohort of patients with PCD included in this study exhibited functional abnormalities on V/Q scans. The adult patients showed more extensive and severe defects as compared with pediatric patients. The physiological impairments were accompanied by an age-related decline in FEV_1_ . Furthermore, there was increased bronchiectasis severity with age on chest CT scans.

The imaging abnormalities observed in this study reflect the underlying mechanism of PCD. The dysfunctional ciliary motility leads to chronic mucus accumulation, which increases the risk of recurrent infections, progressive airway changes, and remodeling [ 10 ]. Several mechanisms affect PCD, including mucus plugging, atelectasis, and bronchial wall damage, all of which may result in regional ventilation defects and localized hypoxic vasoconstriction, causing secondary perfusion defects [ 18 ]. These mechanisms were evident in our V/Q findings, where ventilation abnormalities appeared early in pediatric patients. The perfusion defects became more pronounced in adults with parallel structural damage seen on chest CT scans. Previous studies have shown that V/Q mismatch is a sensitive indicator of regional lung dysfunction; examples include diseases such as cystic fibrosis [ 19 ] and chronic obstructive pulmonary disease [ 20 ], in which heterogeneous lung involvement is common.

The findings in V/Q abnormalities across patients with PCD, and their correlation with age and FEV_1_ , support the potential role of V/Q scans as a tool for future monitoring of PCD progression. The V/Q scans may detect physiologic impairment before gross structural changes become evident on chest CT scans [ 21 ], particularly in pediatric patients. In our cohort, pediatric patients with relatively preserved spirometry already exhibited patchy ventilation defects. These findings explore the possible utility of V/Q scans in identifying early stages of disease, which aligns with MRI-based studies in PCD showing that ventilation and perfusion abnormalities can precede spirometric decline [ 22, 23]. The identification of regional functional impairment by V/Q scans may help PCD providers tailor personalized interventions. These may include intensifying airway clearance strategies and counseling adults with extensive V/Q defects about early referral for lung transplant evaluation.

In cystic fibrosis literature, perfusion abnormalities on V/Q scans have been shown to correlate with disease severity and prognosis, with greater disparity predicting poorer outcomes [ 24 ]. Furthermore, in COPD, V/Q scans reveal disease heterogeneity and guide surgical planification for volume reduction and other interventional therapy [ 25 ]. Nevertheless, in PCD, the use of V/Q scans is not included in recent PCD guidelines. While radioaerosol clearance studies have proven valuable for diagnosing ciliary dysfunction [ 26 ], few studies have examined the use of standard V/Q scans to evaluate lung function or progression. The findings presented in this study begin to fill this knowledge gap by characterizing typical V/Q scan patterns in genetically defined patients with PCD and demonstrating their relationship with clinical and structural markers of disease currently used in clinical practice.

This study has several notable strengths. Our study focuses on a well-defined genetic subgroup of PCD with a single pathogenic genetic variant. The uniformity presented in this study minimizes genetic heterogeneity as a confounding factor. The results allow for more precise interpretation of clinical, functional, and imaging findings of this PCD cohort. The comparison of different variables, including chest CT imaging and FEV_1_, offers a complementary perspective on PCD severity. The inclusion of pediatric and adult patients across different clinical presentations provides age-related trends in pulmonary function decline. Also, structural damage and physiologic impairment, particularly in relation to chronic infection status with *P. aeruginosa* or other PCD-related pathogens was explored. This characterization enhances the relevance and interpretability of our findings within the context of genotype-specific disease progression in PCD. Apart from the sample size and the possible generalizability of the findings, the absence of semi-quantitative scoring for V/Q abnormalities and the lack of longitudinal follow-up precluded the assessment of temporal changes or treatment response at the individual level. Future studies may use quantitative methods to identify statistical differences and validate results in cohorts with PCD genetic diversity.

The possible integration of V/Q scans with advanced functional imaging modalities, such as hyperpolarized gas MRI or dynamic V/Q MRI, may offer additional physiological insights into regional lung dysfunction in patients with PCD. Each multimodal approach in physiologic studies and imaging could ultimately support the development of precision medicine strategies in PCD. The final goal should be to enable genotype-informed treatment selection and dynamic monitoring of disease progression and therapeutic response over time.

## CONCLUSION

A progressive age-related physiological and anatomical lung impairment was observed in patients with PCD carrying the homozygous *RSPH4A* [c.921+3_921+6 delAAGT] genetic variant. This analysis revealed a possible pattern of declining FEV_1_, increasing bronchiectasis on chest CT scans, and worsening V/Q mismatch on scans. Moreover, V/Q scans were able to identify physiological abnormalities even in children with relatively preserved spirometry. These findings highlight the potential utility of V/Q scans as a tool for regional lung dysfunction detection. V/Q scans may support the role of current imaging modalities and PFTs in the longitudinal monitoring of PCD progression. Additional prospective studies are needed to define its prognostic value and potential to guide personalized clinical management in PCD.

## Figures and Tables

**Table 1:** Clinical and demographic characteristics: Demographic data, clinical parameters, and imaging findings of ten patients with PCD.

**Patient ID**	**Age (years)**	**Sex**	**FEV_1_ (%)**	**Infection Status**	**Bronchiectasis (chest CT scan)**	**V/Q Scan Findings**
PCD-01	11	Male	48	*B. cepacia*	Moderate	Patchy lower lobe defects
PCD-02	12	Male	66	*H. parainfluenzae*	Mild	Mild peripheral defects
PCD-03	15	Female	73	*P. aeruginosa*	Mild	Moderate mismatch
PCD-04	18	Female	66	*P. aeruginosa*	Moderate	Moderate mismatch
PCD-05	20	Female	58	*P. aeruginosa*	Moderate	Moderate mismatch
PCD-06	34	Female	39	*P. aeruginosa*	Severe	Bibasilar defects
PCD-07	40	Female	42	*P. aeruginosa*	Severe	Bibasilar defects
PCD-08	42	Female	53	*P. aeruginosa*	Severe	Bibasilar defects
PCD-09	55	Female	51	*P. aeruginosa*	Severe	Severe mismatch
PCD-10	55	Male	32	*P. aeruginosa*	Severe	Severe mismatch

**Note:** The FEV_1_ shows a trend of declining values with age. *Pseudomonas aeruginosa* predominates among adults. Younger patients with PCD presented with other organisms such as *Burkholderia cepacia* and *Haemophilus parainfluenzae*. Varying degrees of bronchiectasis severity were present, with adult patients exhibiting more structural lung damage than pediatric patients. V/Q scans revealed physiologic abnormalities consistent with V/Q mismatch in all cases.

## Data Availability

The data that support the findings of this study are available from the corresponding author, [G.R.-R.], on special request.

## LIST OF ABBREVIATIONS

PCD: Primary Ciliary Dyskinesia
PFTs: Pulmonary Function Tests
V/Q: Ventilation/Perfusion
CT: Computed Tomography
*RSPH4A*: Radial Spoke Head Component 4A
*B. cepacia*: Burkholderia cepacia
*H. parainfluenzae*: Haemophilus parainfluenzae
*P. aeruginosa*: Pseudomonas aeruginosa

## STANDARDS OF REPORTING

STROBE guidelines were followed.
